# N-Methyl-D-aspartate receptors: structure, signaling and roles in atherosclerosis

**DOI:** 10.1186/s12967-026-08399-1

**Published:** 2026-06-06

**Authors:** Jing Cai, Zi-qiang Luo, Min Zhang, Zhen-wang Zhao, Lu Ren, Hai-peng Cheng

**Affiliations:** 1 Department of Pathology, The Second Xiangya Hospital, Central South University, Changsha, Hunan 410011 China; 2 Department of Physiology, Xiangya School of Medicine, Central South University, Changsha, Hunan China; 3 Key Laboratory for Arteriosclerology of Hunan Province, Institute of Cardiovascular Disease, Department of Bioinformatics and Medical Big Data, Hengyang Medical School, Hunan International Scientific and Technological Cooperation Base of Arteriosclerotic Disease, University of South China, Hengyang, Hunan 421001 China; 4 School of Basic Medicine, Health Science Center, Hubei University of Arts and Science, Xiangyang, 441053 China; 5 Department of Dermatology, The Second Xiangya Hospital, Central South University, Changsha, Hunan 410011 China; 6 Hunan Clinical Medical Research Center for Cancer Pathogenic Genes Testing and Diagnosis, Changsha, Hunan 410011 China

**Keywords:** N-Methyl-d-aspartate receptors, Atherosclerosis, Lipid metabolism, Inflammation, Macrophage, Vascular smooth muscle cells phenotype switching, Endothelial dysfunction

## Abstract

Atherosclerosis, a chronic inflammatory disease of the arterial wall, is a leading cause of cardiovascular diseases worldwide. The complex pathogenesis of atherosclerosis involves genetic predisposition, environmental factors, and immune responses. N-Methyl-d-aspartate receptors (NMDARs), a subclass of glutamate receptors, are critical for synaptic plasticity, learning, and memory in the central nervous system (CNS). Non-neuronal NMDARs are poorly understood compared to neuronal receptors, but there is a developing consensus that they have distinct structural and functional properties when activated by glutamate and NMDARs co-agonists. Emerging evidence indicates that non-neuronal NMDARs may participate in an array of physiological and pathophysiological processes, including but not limited to driving macrophage polarization, lipid dysregulation in macrophages, inflammation response, vascular smooth muscle cells phenotype switching and endothelial dysfunction, thereby fueling atherogenesis. This review discusses the association between NMDARs genes and atherosclerosis risk, molecular mechanisms underlying NMDARs-mediated regulation of atherosclerosis-related cells, and potential therapeutic implications. Besides, we introduce some pharmacological tools that can be used for studying NMDARs outside the CNS, which reflect modern subunit-selective agents to provide more precise insight into NMDARs mediate the various effects. Overall, the study of NMDARs may provide insights into the pathogenesis of atherosclerosis and lead to the development of more effective therapeutic strategies.

## Introduction

Atherosclerosis, a chronic inflammatory vascular disease, affects arteries throughout the body. Characterized by the build-up of fatty deposits within the arterial walls, known as plaques [1], atherosclerosis is a significant global health burden as a major cause of heart attacks and strokes [2]. The process begins with the accumulation of low-density lipoprotein (LDL) particles in the arterial walls, where LDLs undergo oxidative modifications, such as esterification and lipid peroxidation. Oxidized low-density lipoproteins (oxLDLs) recruit immune cells, particularly monocytes, which migrate from the blood to the arterial wall. Here, they transform into macrophages and, after engulfing oxLDLs, foam cells, thereby contributing to lipid accumulation [3]. Simultaneously, endothelial cells become dysfunctional, leading to the production of molecules that recruit more immune cells. As atherosclerosis progresses, the unstable plaques can rupture, exposing the necrotic core, triggering thrombus formation, blocking arteries, and leading to a heart attack or stroke [4, 5]. 

Glutamate (Glu), one of the most abundant amino acids, plays a crucial role in nutrition, metabolism, and signal transduction, in addition to its function in protein structure [6]. In the central nervous system (CNS), Glu is an important excitatory neurotransmitter, although excess Glu can cause cell death. The role of Glu in peripheral tissues has recently gained increased research attention [7]. However, the potential impact of Glu-mediated neurotoxicity on atherosclerosis remains largely unknown. In mammals, there are two types of Glu receptors: ionotropic and metabotropic. N-Methyl-d-aspartate receptors (NMDARs), a class of ionotropic Glu receptors, are involved in vital physiological functions, such as inflammatory responses, oxidative stress, and apoptosis by binding to Glu and inducing postsynaptic depolarization, which mediates calcium entry [8]. The unique dual-gating mechanism of NMDARs, involving voltage-dependent magnesium block and ligand binding, regulates neuronal excitability. NMDARs play crucial roles in human neuronal development and survival, synaptic plasticity, learning, memory, pain perception, brain circuit formation, and cell survival [9, 10]. 

Central NMDARs are important for diverse physiological processes, primarily the modulation of excitatory synaptic transmission and synaptic plasticity [11]. These receptors are intricately linked to a wide array of diseases, including neurological conditions, such as Alzheimer’s disease, Parkinson’s disease, Huntington’s disease, and schizophrenia [12]. However, a recent discovery has shown that NMDARs are present in the CNS as well as in peripheral tissues [13]. Currently, the role of NMDARs in atherosclerosis is poorly understood. Acute and chronic activation of NMDARs can lead to elevated blood pressure and tachycardia, which are associated with increased susceptibility to arrhythmias [14, 15]. Both myocardial ischemia and ischemia-reperfusion can induce ventricular fibrillation and ventricular tachycardia. These symptoms can be improved by using NMDARs antagonists, such as MK-801, 6-cyano-7-nitroquinoxaline-2,3-dione (CNQX), ketamine, or memantine [16]. In addition, a recent clinical study reported that the NMDARs antagonist amantadine, when used to treat patients with Parkinson’s disease, is associated with reduced risks of all-cause mortality, myocardial infarction, cerebral infarction, heart failure, and arrhythmias [17]. This indicates that NMDARs antagonists may have potential therapeutic value in cardiovascular diseases. This review summarizes the current understanding of the role of peripheral NMDARs in cells associated with atherosclerosis and potential effects of these receptors on vascular function, endothelial dysfunction, smooth muscle cell proliferation, inflammation, and lipid metabolism. In addition, by identifying gaps in our knowledge, this review serves as a reference for future research endeavors.

## Genes and structures

Mammals possess homotetrameric NMDARs composed of subunits encoded by three gene families: NMDAR1 (GluN1 or Grin1), NMDAR2 (GluN2 or Grin2), and NMDAR3 (GluN3 or Grin3) [18]. Although NMDAR1 is encoded by a single gene, its transcripts can produce at least eight different variants through alternative splicing. The complex composition of different subunits and splice variants constitutes the primary basis for the functional diversity of NMDARs [19, 20]. In contrast, the GluN2 subunits are composed of four different isoforms, each encoded by a separate gene: GluN2A, GluN2B, GluN2C, and GluN2D [21]. The GluN3 family also consists of two genes that encode two different isoforms: GluN3A and GluN3B [22, 23]. 

**Fig. 1:**
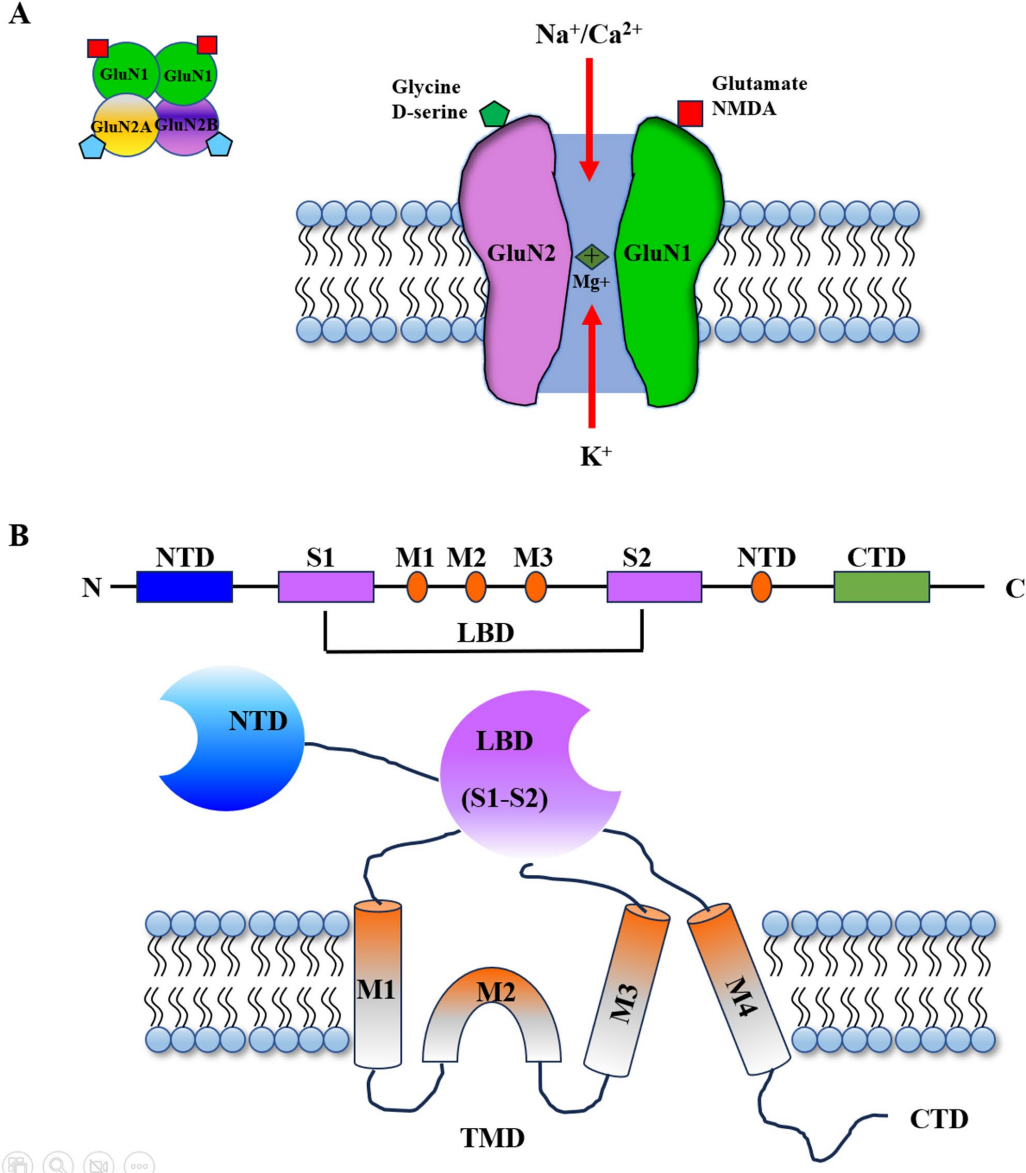
Structure and domain organization of N-methyl-d-aspartate receptors (NMDARs). (**A**): Structure of the NMDAR. NMDARs are heterotetramers comprising two obligatory GluN1 subunits and two GluN2 subunits. The GluN1 subunit contains the agonist-binding site (glutamate and NMDA), whereas the GluN2 subunit contains the glycine (or serine) binding site. NMDARs are permeable to intracellular K^+^ (outward direction) and extracellular Na^+^ and Ca^2+^ (inward direction). Within the NMDAR channel pore, there is an Mg^2+^ binding site. For the NMDAR to be activated by glutamate, Mg^2+^ in this site must first be released, a process typically triggered by AMPA-mediated membrane depolarization. (**B**): Linear representation of the subunit polypeptide chain and schematic illustration of the subunit topology. NMDAR subunits encompass two extracellular domains (the N-terminal domain (NTD) and the ligand-binding domain (LBD)), a transmembrane domain (TMD) that constitutes a segment of the ion channel pore, and an intracellular C-terminal domain (CTD). The LBD is delineated by two stretches of amino acids known as segments S1 and S2. Within the TMD, three helices traverse the membrane (M1, M3, and M4), accompanied by a loop that re-enters the membrane (M2)

Functional NMDARs are typically formed as heterotetramers composed of two dimers, each consisting of GluN1 and GluN2 subunits (Fig. 1). The GluN1-GluN2 dimer, considered the basic functional organizational structure of NMDARs, contains various sites for the binding and recognition of different ligands with distinct physiological or pharmacological functions. Each GluN1 subunit contains a glycine-binding site, whereas each GluN2 subunit contains a Glu-binding site. Therefore, each NMDAR comprises of two glycine- and two Glu-binding sites [24–26]. Each subunit of this ionotropic receptor has a highly conserved molecular structure divided into four functional domains: the N-terminal extracellular, ligand-binding, pore-forming transmembrane (TMD), and carboxy-terminal intracellular (CTD) domains [27]. The TMD is formed by three transmembrane helices (M1, M3, and M4) and one re-entrant loop (M2), which are hydrophobic and play a role in pore opening (i.e., channel gating). The M2 segment penetrates the membrane to form an ion channel, whereas the extracellular region of the central ion channel pore is formed by elements of the third transmembrane segment [28]. In addition to the natural glycine and Glu binding sites in the GluN1- GluN2 dimer, the extracellular region of NMDAR2 contains specific binding sites for endogenous ligands that serve as redox sites for protons and zinc. These endogenous ligands can regulate NMDARs activity by increasing or decreasing calcium flux through the receptor under physiological or pathological conditions, respectively [29, 30]. Exogenous ligands, such as steroids, ethanol, and ifenprodil, and synthetic molecules may serve as experimental tools for analyzing the properties of NMDARs and aiding the development of antagonists with therapeutic potential.

**Fig. 2:**
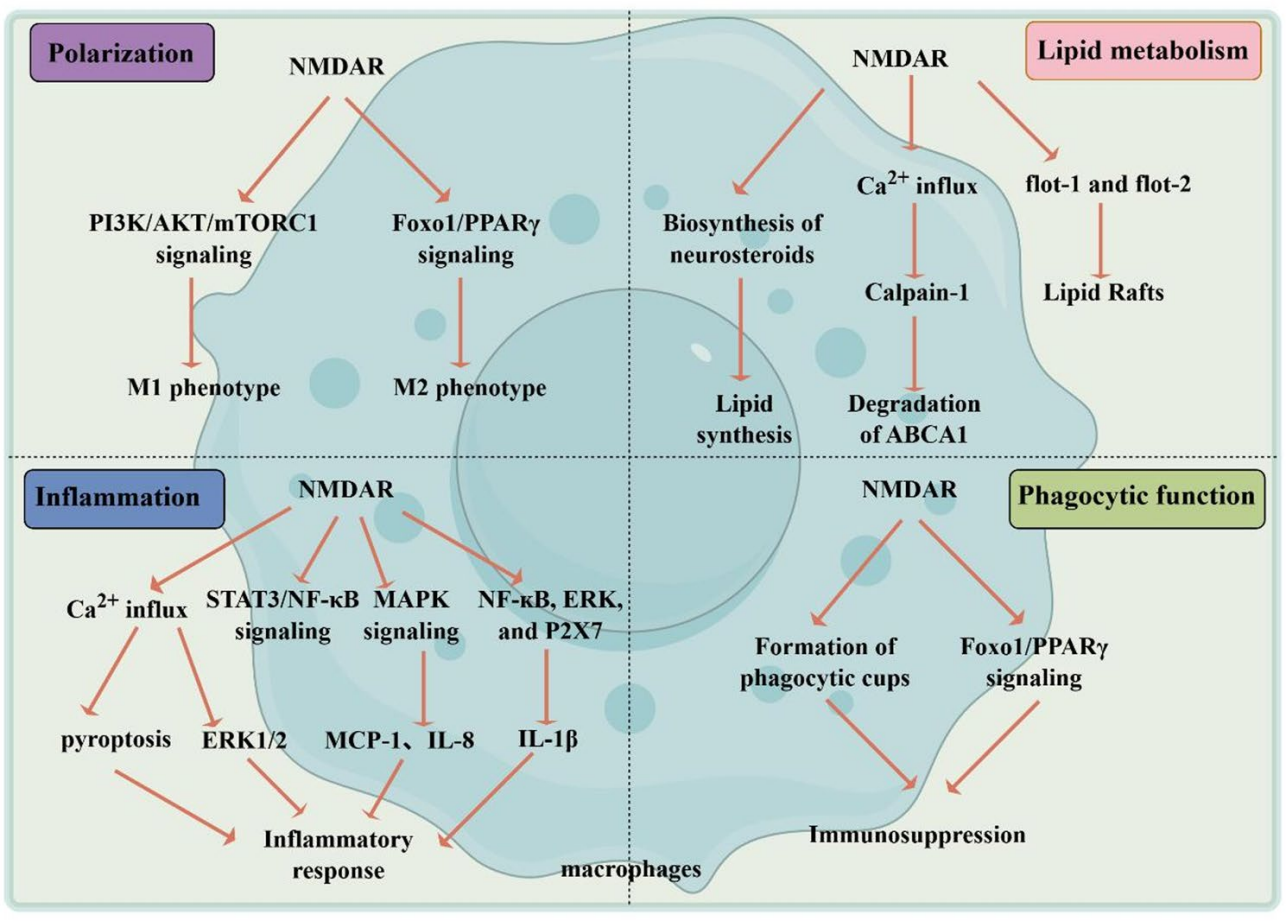
Potential role of N-methyl-d-aspartate receptors (NMDARs) in atherosclerosis (AS). In macrophages, NMDAR activation leads to macrophage polarization into M1 and M2 phenotypes, affecting inflammation and the immune response. The PI3K/AKT/mTORC1 signaling pathway is implicated in glycolysis and lipid metabolism. Furthermore, Ca^2+^ influx triggers pyroptosis and the activation of inflammatory pathways involving NF-κB, ERK1/2, and the NLRP3 inflammasome. The ERK1/2/PPARα pathway and Ca^2+^ influx is associated with neurosteroid biosynthesis and the degradation of ABCA1, leading to lipid accumulation. PI3K, phosphatidylinositol 3-kinase; AKT, protein kinase B; mTORC1, mammalian target of rapamycin complex 1, Foxo1, Forkhead box O1; PPAR, peroxisome proliferator-activated receptor; ERK1, extracellular regulated kinase 1; ABCA1, ATP-binding cassette transporter A1; flot-1/2, flotillin 1/2; ASC, apoptosis-related dot-like protein ASC; NLRP3, NLR family pyrin domain containing 3; NF-κB, nuclear factor kappa-B; MAPK, mitogen-activated protein kinase; STAT3, signal transducer and activator of transcription 3; ROS, reactive oxygen species; MCP-1, monocyte chemoattractant protein-1; IL-8, interleukin 8; P2 × 7, purinergic receptor P2X, ligand-gated ion channel 7

**Fig. 3:**
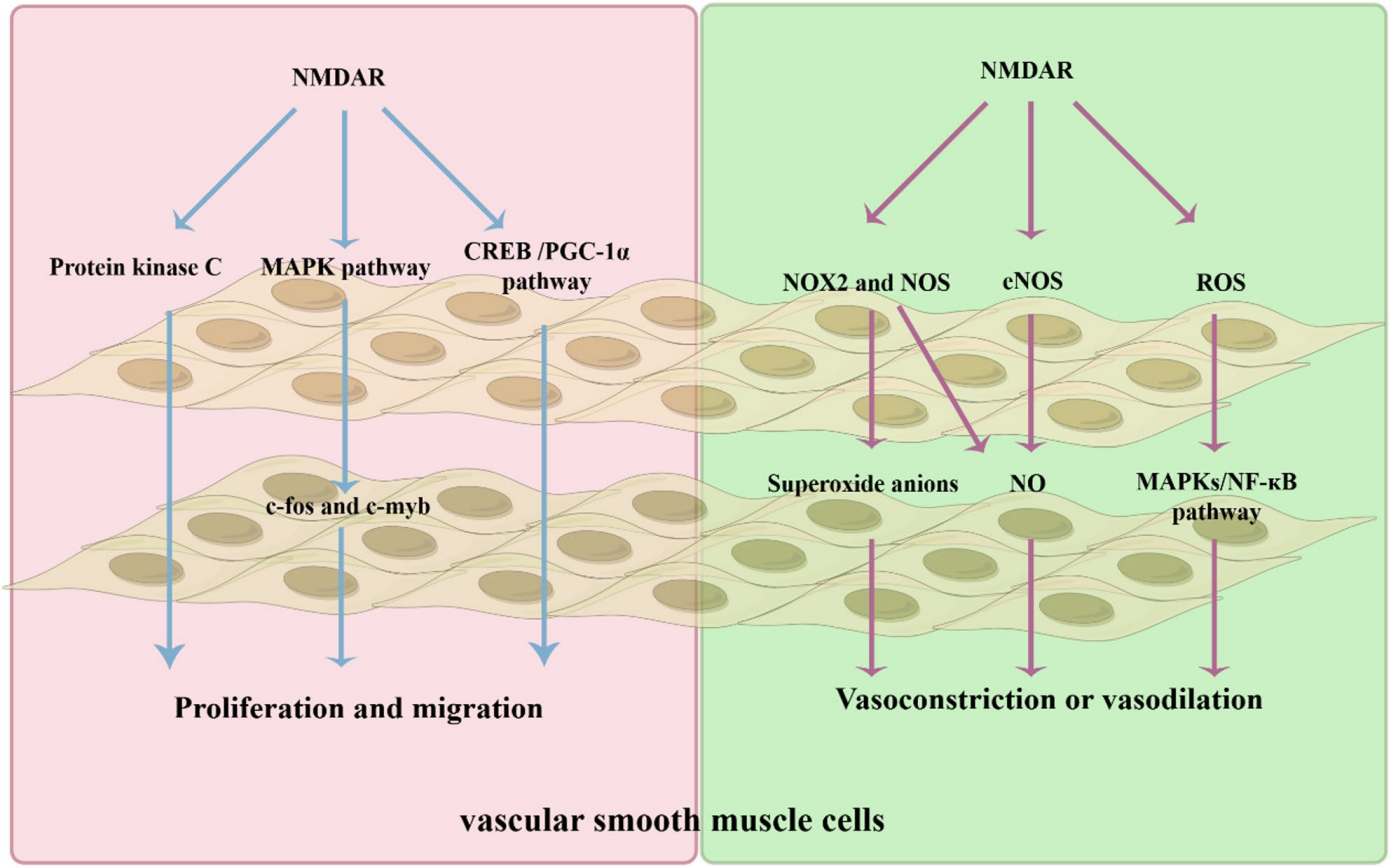
Potential role of N-methyl-d-aspartate receptors (NMDARs) in atherosclerosis (AS). NMDARs in vascular smooth muscle cells affect cell proliferation and migration and the balance between vasoconstriction and vasodilation. Protein kinase C, c-Fos, and c-Myb, along with the MAPK pathway, are involved in the regulation of blood flow and pressure. CREB, cAMP-response element binding protein; PGC-1α, peroxisome proliferator-activated receptor γ coactivator 1α; ROS, reactive oxygen species; NF-κB, nuclear factor kappa-B; MAPK, mitogen-activated protein kinase; NOX2, NADPH oxidase 2; NOS, nitric oxide synthase; NO, nitric oxide

**Fig. 4:**
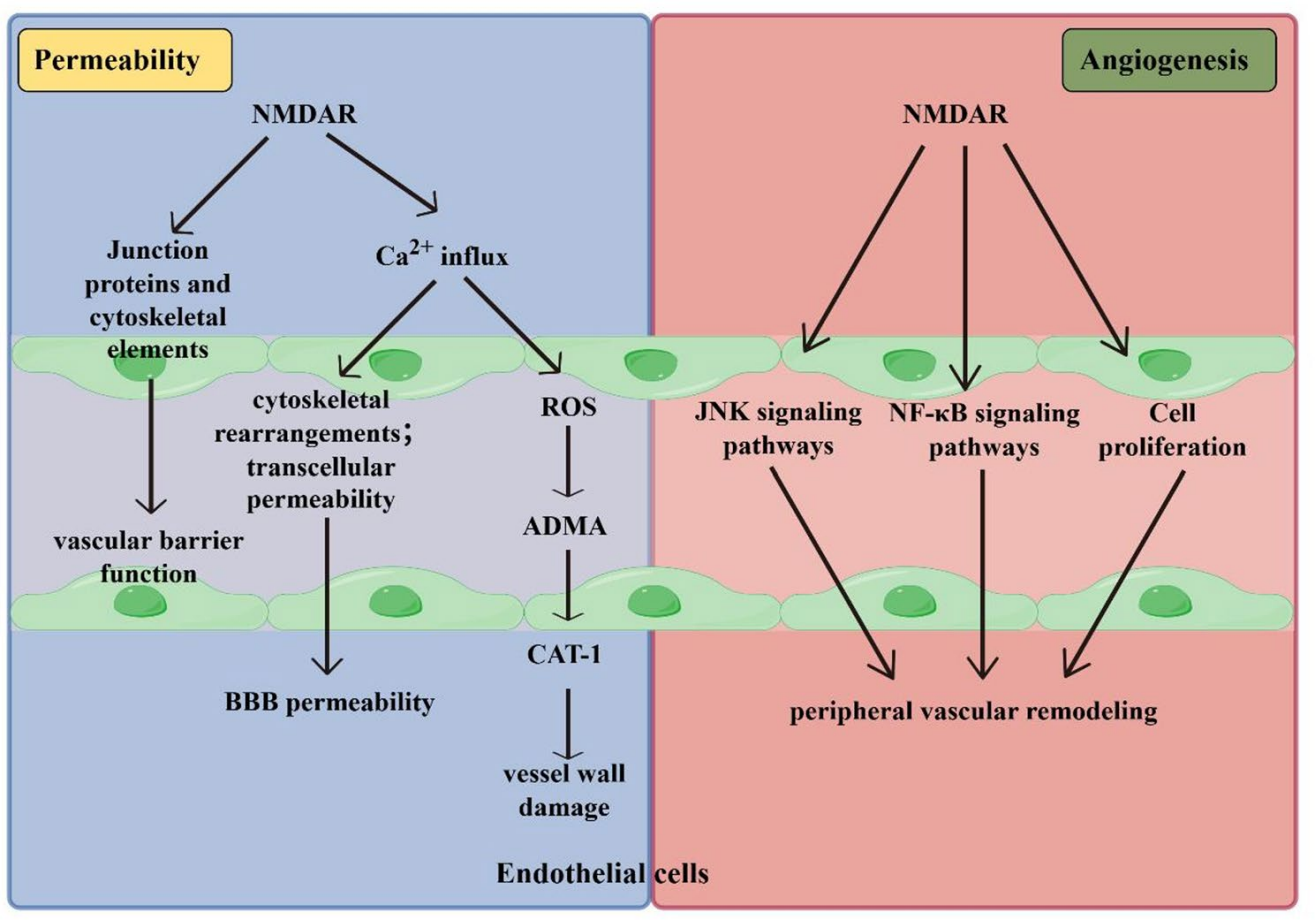
Potential role of N-methyl-d-aspartate receptors (NMDARs) in atherosclerosis (AS). In endothelial cells, NMDARs affect permeability, angiogenesis, and vascular barrier function. Ca2+ influx and ROS production via NOX2 and NOS contribute to vessel wall damage and alterations in blood–brain barrier (BBB) permeability. Peripheral vascular remodeling is regulated by the JNK and NF-κB signaling pathways. ROS, reactive oxygen species; ADMA, asymmetric dimethylarginine; CAT-1, cationic amino acid transporter 1; JNK, Jun N-terminal protein kinase; NF-κB, nuclear factor kappa-B; NR1, N-methyl-d-aspartate receptor 1

## Synthesis and transportation

The endoplasmic reticulum (ER) is the primary organelle in which protein and lipid trafficking begins. Membrane proteins, with complex structures and large molecular weights, must be correctly folded and assembled into tertiary and quaternary structures before they can be transported to the plasma membrane to exert their physiological functions [31]. NMDARs biogenesis begins with the transcription of subunit genes, specifically GRIN genes, which encode the GluN1 subunit. The expression and translation of the GluN1 subunit surpasses those of the GluN2 and GluN3 subunits [32]. Unassembled monomers of the GluN1 and GluN2 subunits exist in the ER and are assembled on demand to form tetrameric NMDARs [33]. Currently, it is widely accepted that homodimeric pairs of GluN1-GluN1 and GluN2-GluN2 subunits first form and then assemble to form heterotetrameric receptors [33]. Once the protein is fully folded, the tetrameric NMDARs is transported to the ER-Golgi intermediate compartment (ERGIC) and Golgi apparatus for processing [34]. The NMDARs subunit interacts with scaffolding proteins in the ER, such as synapse-associated protein 102, through the PSD95/Dlg1/ZO-1 domain, which allows its release from the ER and downstream trafficking [35, 36]. 

Lichnerova et al. have shown that two glycosylation sites within the GluN1 subunit, Asn 203 and Asn 368, are essential for the anterograde trafficking of NMDARs, whereas glycosylation sites within the GluN2 subunit do not affect receptor trafficking [37]. In addition, another post-translational modification, palmitoylation, is involved in the anterograde trafficking of NMDARs. The two cysteine clusters in the CTD of GluN2A and GluN2B are palmitoylated. When the first cysteine residue in the proximal region of the membrane is palmitoylated, the surface expression of NMDARs increases, whereas when the second cysteine residue in the CTD is palmitoylated, NMDARs accumulate in the Golgi apparatus, leading to decreased NMDARs expression [38]. Moreover, mutations in the cysteine cluster in the membrane-proximal region result in the decreased surface expression of NMDARs [39]. 

During anterograde trafficking, NMDARs can reach the plasma membrane through classical or non-classical pathways. In the classical pathway, NMDARs follow the secretory pathway of the neuronal somatodendritic compartments, through which the protein leaves the ER and enters the ERGIC. Once in the Golgi apparatus, the receptor can be inserted into vesicles for transport to the plasma membrane; alternatively, the vesicles can be transported on microtubule cytoskeletal elements to the dendrites [40]. In the non-classical pathway, NMDARs interact with the glutamatergic synapse scaffolding protein SAP97 and calcium/calmodulin-dependent serine protein kinase to bypass ERGIC and traffic directly to the Golgi apparatus, where they are transported in vesicles to presynaptic terminals [41, 42]. A recent study has shown that the DISC1 scaffold protein plays an important role in the trafficking of NMDARs within the membrane, promoting the insertion of NMDARs into the plasma membrane and their trafficking into synaptic clefts [43]. 

## NMDARs signaling

Signal transduction mediated by NMDARs has been extensively studied and well-established in the CNS; [44] however, corresponding research in peripheral tissues is still in the early stages of development and continuous growth. Because our understanding of the signaling molecules mediating cardiovascular responses of peripheral NMDARs is merely the tip of the iceberg, we will broadly outline the potential common signaling networks regulating peripheral and central NMDARs activation and their biological functions.

### Non-neuronal glutamate

Glutamate is a crucial excitatory amino acid mediator of synaptic transmission in the CNS. Following regulated vesicular release from axon terminals, synaptic glutamate diffuses freely and activates an array of presynaptic, postsynaptic, and/or cell-surface receptors [18]. Aside from nervous system tissues, extracellular glutamate in the vascular microenvironment to activation of NMDARs play an essential role for many biological processes. In the context of atherosclerosis and the vascular microenvironment, local of non-neuronal glutamate are primarily derived from the contributions of basic metabolic reactions, such as deamination and gluconeogenesis, muscle reserve (released during exercise or injury) [45], platelet dense-granule secretion (high-affinity EAAT-like transporter) [46, 47], Leukocyte and endothelial synthesis/release [48] contribute to the pool of non-neuronal glutamate. Besides, dietary protein, including meat, fish, beans, etc. [49]., acts as a systemic background source that influences baseline plasma levels.

### Calcium signaling

Calcium signaling and NMDARs are intricately linked in the nervous system, playing crucial roles in synaptic function and plasticity [50]. NMDARs, which are calcium-permeable ion channels, allow calcium influx upon activation by glutamate and glycine, and this influx is essential for triggering intracellular signaling cascades. This calcium entry can activate enzymes such as calmodulin and CaMKII, influencing synaptic structure and function [51]. Within the cardiovascular system, the activation of NMDARs triggers a series of complex intracellular signaling pathway changes. When NMDARs is activated, the intracellular calcium ion level significantly increases, leading to calcium overload. This state of calcium overload activates a variety of calcium-dependent enzymes, including phospholipase, protein kinases, and proteases [7]. These enzymes initiate a series of cellular damage reactions: phospholipase degrades membrane phospholipids, compromising the integrity of the cell membrane; protein kinases and proteases lead to protein denaturation, affecting the normal function of cells [7]. Furthermore, calcium overload also leads to an increase in the production of reactive oxygen species (ROS), further exacerbating cellular oxidative stress damage. Mitochondria, which serve as the powerhouse of the cell, are also severely affected by calcium overload. Excessive calcium ions entering the mitochondria lead to mitochondrial dysfunction, which in turn affects ATP production. ATP is the energy source for cells, and its reduction weakens the survival capacity of the cell, ultimately leading to cell apoptosis or necrosis [52, 53]. 

### Nitric oxide

NO and NMDARs are closely interconnected in the nervous system, with NO playing a crucial role in modulating NMDARs activity through multiple mechanisms [54]. Activation of NMDARs leads to calcium influx and PKC mediated activation of neuronal nitric oxide synthase (nNOS) to produce NO [55]. NO can directly modify NMDA receptors through S-nitrosylation, either enhancing or inhibiting their function [56] The role of NO in the cardiovascular system is still being studied. NO exerts its vasodilatory, antiplatelet, antithrombotic, and anti-inflammatory properties within the vascular system [57]. Endothelial (e)NOS, nNOS, and inducible (i)NOS generates NO from oxygen and L-arginine, activating guanylate cyclase to convert GTP to cGMP, which promotes vasodilation [58]. Under normal conditions, NO exerts anti-atherosclerotic effects through multiple pathways. These include reducing endothelial cell apoptosis and activation by inhibiting nuclear factor-κB (NF-κB) and the expression of inflammatory genes [59]. In addition, NO can inhibit atherosclerosis by attenuating the adhesion interactions between leukocytes and endothelial cells [60]. Matrix metalloproteinases (MMPs) are believed to promote atherosclerosis [61]. Sodium nitroprusside, an NO donor, increases NO production in HMCL cells, which can combine with superoxide to form peroxynitrite, thereby activating MMPs [62]. A previous clinical study indicated that in patients with hypercholesterolemia, the activity of endothelial nitric oxide synthase (eNOS) and the expression of NOS protein in platelets are also significantly reduced [63]. 

### Reactive oxygen species (ROS)

Overactivation of NMDA receptors triggers calcium influx, which activates enzymes such as NADPH oxidase, leading to the production of reactive ROS, including superoxide, hydroxyl radicals, and hydrogen peroxide. These ROS can cause oxidative stress, damage cellular components, and induce neurotoxicity [64]. Excessive ROS production is also associated with neuroinflammation and neurodegenerative diseases such as Alzheimer’s disease, where ROS exacerbate neuronal dysfunction and death [65]. Brennan et al. investigated that NADPH oxidase (NOX) contributes to superoxide production triggered by NMDARs in cultured neurons and the mouse hippocampus [66]. NMDARs activation boosts levels of NOX2 and NO, which together enhance ROS production in the CNS by activating ERK1 [67]. Similar observations were made in cardiovascular tissues, where peripheral NMDARs activation also drives ROS production. Despite the widespread presence of various NOX subtypes in cardiovascular tissues [68], their role in peripheral NMDARs-mediated ROS production and its functional significance had remained unclear.

## NMDARs and atherosclerosis-related cells

### Overview

Atherosclerosis is a chronic inflammatory disease that involves various types of cells and their interactions, including:

- Monocytes, which migrate from the bloodstream to the damaged endothelium and differentiate into macrophages. These cells play a key role in inflammation and clearance during atherosclerosis. They engulf oxLDLs, forming foam cells that are characteristic of atherosclerotic plaques.
- VSMCs, which normally maintain the structure and function of blood vessels. In atherosclerosis, these cells transition from a contractile to a synthetic state, migrate to the intima, and proliferate to form the main body of the plaque.
- Endothelial cells forming the vascular endothelium, which serves as a barrier between blood and the vessel wall. Dysfunction of the vascular endothelium is the initial step in the development of atherosclerosis. Endothelial cells release factors that regulate vascular dilation, inflammation, and coagulation. These cells interact and collectively drive the progression of atherosclerosis by secreting various bioactive molecules, such as cytokines, growth factors, chemokines, and enzymes. The role of NMDARs in cells implicated in atherosclerosis and its correlation with the development and progression of atherosclerosis are provided in Figs. 2, 3 and 4.

### NMDARs and macrophages

#### Overview

Macrophages, a heterogeneous population of immune cells that play pivotal roles in host defense and tissue homeostasis, have been found to express NMDARs, promoting a novel frontier in the understanding of their complex regulatory mechanisms. NMDARs activation by Glu or other agonists triggers the influx of calcium ions, which act as secondary messengers to activate various downstream signaling pathways. These pathways regulate macrophage activation, polarization, cytokine production, and phagocytic function. The functions of NMDARs in macrophages are listed in Table 1.

**Table 1:** NMDARs diversity in the cardiovascular system

Cell or tissue	Subunit expression	RNA analysis	Proposed function	Refs
Macrophages	GluN1; GluN2A-D; GluN3A, 3B	++++	Polarization, lipid metabolism, and inflammation	[72, 73, 80, 86, 135, 70, 136]
Vascular smooth muscle cells	GluN1; GluN2A-D	+/++	Proliferation and migration, vasoconstriction, and vascular remodeling	[106–108]
Endothelial cells	GluN1; GluN2A-D	+	Proliferation and migration, angiogenesis, and apoptosis	[119, 183]
Heart (atrium and ventricle)	GluN1; GluN2A, 2B, 2D	+++	Mitochondrial dysfunction, oxidative stress, and apoptosis	[77, 184–186]
Vasculature (pulmonary artery and aorta)	GluN1	+++	Not investigated	[187, 188]

NMDARs, N-methyl-d-aspartate receptors, More “+” signs indicate a stronger level

#### NMDARs and macrophage activation

Macrophage activation is closely associated with atherosclerosis. Macrophages modify the uptake of LDL, leading to the formation of foam cells, an early event in atherosclerosis. Traditionally known for their phagocytic and antigen-presenting functions, macrophages have been shown to express NMDARs. This receptor is located on the cell surface as well as intracellularly, pointing to its involvement in membrane-bound and intracellular signaling pathways [69]. Cheng et al. demonstrated that the NMDARs-specific agonist NMDA robustly activates surface NMDARs on primary bone-marrow macrophages and THP-1-derived macrophages, eliciting Ca²⁺ influx and downstream calpain signaling [70]. Quinolinate (QA) drives macrophage polarization toward an M2-like phenotype by activating NMDARs and the Foxo1/PPARγ signaling axis [71]. Tissue-type plasminogen activator (tPA) is the chief intravascular trigger of fibrinolysis and doubles as a ligand for cell-surface signaling receptors. Elisabetta et al. show that macrophages express NMDARs, a receptor required for anti-inflammatory signaling and for tPA-driven modulation of cytokine expression, thereby coupling the fibrinolytic system to innate immunity [69]. Besides, the expression of NMDARs subtypes is high in alveolar macrophages (AMs) of newborn and adult rats. Glu plays a role in hyperoxic-induced lung injury in newborn rats through NMDARs [72]. NO, a small molecule produced by the NOS family of enzymes, plays a crucial role in the regulation of vascular tone, platelet aggregation, and immune responses. NO modulates macrophage activity, affecting phagocytosis, antigen presentation, and cytokine production. Shang et al. reported that the activation of NMDARs promotes the secretion of NO and other inflammatory mediators by enhancing the activity of inducible NOS in AMs, thereby facilitating the development of lung injury [73]. Although AMs offer a valuable model of NMDARs-mediated macrophage activation, their phenotype differs from intimal macrophages and findings must be interpreted with caution when extrapolated to atherosclerosis. Together, further investigation is required to delineate the precise function of NMDARs in the activation of macrophages and their contribution to the process of atherosclerosis (Fig. 2).

#### NMDARs and macrophage polarization

Macrophages are crucial components of the innate immune system and play vital roles in the defense against pathogens, clearance of dead cells, and maintenance of tissue homeostasis [74]. However, their functionality is not static. Instead, they can undergo polarization, through which they acquire distinct functional phenotypes in response to specific stimuli in their microenvironment. Macrophage polarization, a highly dynamic and plastic process, is affected by a wide range of factors, including cytokines, chemokines, hormones, and other soluble mediators [75]. Two primary macrophage phenotypes, M1 (classically activated) and M2 (alternatively activated) [76], have been identified, although these phenotypes represent the two extremes of a spectrum. Macrophage polarization, a tightly regulated process involving complex signaling pathways and transcriptional programs, has important implications in diverse diseases, including infectious diseases, autoimmune diseases, cancer, and cardiovascular diseases [77]. M1 and M2 macrophages are closely related to the progression of atherosclerosis through polarization between M1 and M2-like macrophages and variations in related metabolic pathways [78]. 

M1 macrophages produce pro-inflammatory cytokines that can promote the development of atherosclerosis, whereas M2 macrophages have anti-inflammatory and immunomodulatory effects that may help mitigate inflammatory responses. Glycolysis, a cellular respiratory metabolic pathway, plays an important role in macrophage polarization and functional changes. The activities of key enzymes related to glycolysis, such as hexokinase, phosphofructokinase, and pyruvate kinase M2, are frequently altered when macrophages transition from a quiescent to a polarized state [79]. Lipopolysaccharide (LPS)-stimulated glycolysis, which is characterized by the upregulation of PI3K/AKT/mTORC1 signaling, is triggered by NMDARs-mediated Ca^2+^ accumulation [80]. This intracellular Ca^2+^ signal, a key regulator of various cellular processes, acts as a molecular switch that controls the LPS-stimulated glycolysis cascade. The activation of the PI3K/AKT/mTORC1 signaling pathway results in the phosphorylation of key enzymes and transcription factors, such as hypoxia-inducible factor 1α and lactate dehydrogenase A, that regulate the expression of genes involved in glycolysis. This process ultimately leads to the polarization of macrophages toward the M1 phenotype, which is associated with pro-inflammatory responses and tissue damage [80]. 

The administration of ketamine, a non-competitive glutamatergic NMDARs antagonist, at a subanesthetic dose of 10 mg/kg in mice has profound effects on immune cell populations. It leads to a reduction in circulating classical pro-inflammatory monocytes as well as promotes the emergence of M2 macrophage subtypes in the spleen and CNS [81]. By inhibiting NMDARs activation, the balance may shift toward M2 polarization, potentially reducing inflammation within atherosclerotic plaques. Although the potential of NMDARs inhibition to shift macrophage polarization toward the M2 phenotype is intriguing, the exact mechanisms and clinical implications require further study. The role of macrophage polarization in atherosclerosis is complex and a nuanced understanding of these processes is essential for developing targeted therapies (Fig. 2).

#### NMDARs and cytokine production by macrophages

Cytokines, which are pivotal in the development of atherosclerosis, are secreted by a range of cell types, including immune, endothelial, and smooth muscle cells. They modulate cellular activities and interactions, thereby affecting inflammation, cell growth, cell movement, and apoptosis [82]. The balance between pro-inflammatory cytokines, such as tumor necrosis factor-α, interleukin (IL)-1β, and IL-6, which exacerbate plaque development and vulnerability, and anti-inflammatory cytokines, such as IL-10, which mitigate inflammation and aid in plaque stabilization, is critical. The interplay between these cytokines is essential for preventing acute cardiovascular events resulting from plaque rupture.

Depending on the specific conditions and signaling milieu, NMDARs activation can either promote or inhibit the production of inflammatory cytokines, thereby modulating the overall immune response. Calcium signaling pathways play a significant role in the development and progression of atherosclerosis. These pathways are involved in the activation of inflammatory cells, such as macrophages, and play a role in the oxidation of LDL cholesterol and formation of foam cells. Dysregulated calcium signaling can also lead to apoptosis and necrosis in the arterial wall, contributing to plaque instability and potentially leading to plaque rupture [83–85]. The activation of NMDARs stimulates the calcium signaling pathway, resulting in the generation of a substantial amount of ROS and increased expression of the pro-inflammatory mediator COX-2 in macrophages [86]. Ding et al. demonstrated that the NMDARs antagonist memantine inhibits the activation of the NLRP3 inflammasome and pyroptosis in macrophages by inhibiting Ca^2+^ influx and subsequent ASC oligomerization, thereby alleviating acute lung injury in a sepsis mouse model [87]. The relationship between NMDARs activation and cytokine production by macrophages in atherosclerosis is likely to be complex and may depend on various factors, including the specific microenvironment and presence of other signaling molecules. Further studies are required to fully elucidate these mechanisms (Fig. 2).

#### NMDARs and the phagocytic function of macrophages

Macrophages play a dual role; they phagocytose bacteria and secrete inflammatory and antibacterial factors to alleviate infections. Furthermore, they can efficiently eliminate diseased and damaged cells through programmed cell death, which is crucial for preserving tissue integrity. Under normal conditions, macrophages actively consume and degrade unwanted dead cells, debris, tumor cells, and foreign bodies [88]. However, in atherosclerosis, macrophages may play different roles. Damage to the arterial wall can sometimes lead to bacterial infections, especially in advanced or complicated lesions. In such cases, the antibacterial activity of macrophages may contain local infections and prevent the spread of bacteria in the arterial wall [89]. 

NMDARs activation facilitates the uptake and degradation of target particles by regulating the expression and activity of phagocytic receptors and promoting the formation of phagocytic cups [90, 91]. Yuan et al. revealed that NMDARs are expressed and activated on macrophages within the tumor microenvironment, thereby enhancing their immunosuppressive capabilities and contributing to tumor immune evasion [92]. Moreover, the modulation of macrophage polarization toward an anti-inflammatory phenotype (M2 type) has beneficial effects in atherosclerosis, potentially by reducing inflammation and promoting plaque stability. Peroxisome proliferator-activated receptors (PPARs) are ligand-dependent transcription factors that play important anti-atherosclerotic roles by regulating triglyceride metabolism and macrophage polarization and inhibiting inflammatory signaling pathways. The accumulation of quinolinic acid triggers the NMDARs in macrophages, activating the FOXO1/PPARγ transcription factor signaling pathway. This, in turn, initiates the polarization of macrophages toward an M2-like phenotype, ultimately fostering immunosuppression [71]. In summary, although the antibacterial function of macrophages is crucial for the immune response against infections, their specific role in atherosclerosis may be related to the modulation of inflammation and immune responses within the arterial wall rather than direct pathogen elimination. Further research is needed to fully understand the interplay between macrophages, antibacterial defense, and the development of atherosclerosis (Fig. 2).

### NMDARs and VSMCs

#### Overview

In cardiovascular diseases, the phenotypic transformation of VSMCs involves the weakening of the contractile ability of cells and enhanced proliferation, migration, and secretion capabilities. This phenotypic transformation underlies the occurrence and development of various cardiovascular diseases, such as atherosclerosis and pulmonary arterial hypertension. NMDARs, which act as receptors for homocysteine, mediate the effects of homocysteine on VSMCs and are involved in the development of atherosclerosis. The activation of these receptors triggers an influx of calcium ions into the cells, leading to vasoconstriction. This effect is mediated by the activation of downstream signaling pathways involving calcium-dependent kinases and phosphatases. In contrast, NMDARs activation can also promote vasodilation by stimulating the production of NO, a potent vasodilator. The precise balance between these opposing effects likely depends on the specific conditions and signaling milieu within VSMCs. Table 1 lists the functions of NMDARs in VSMCs.

#### NMDARs and the proliferation and migration of VSMCs

The presence and function of NMDARs in VSMCs are associated with various cardiovascular diseases. NMDARs activation in VSMCs promotes cell proliferation and migration, which are key processes in the development of atherosclerosis. As atherosclerosis progresses, VSMCs migrate from the tunica media to the tunica intima, contributing to atherosclerotic plaque formation. Furthermore, VSMCs are responsible for the production of extracellular matrix components, such as collagen and elastin, which provide structural support for plaques [93]. The effect of homocysteine on neural crest-derived VSMCs proliferation is blocked by MK-801, a non-competitive antagonist of NMDARs, through a reduction in phospholipid production, the inactivation of protein kinase C, and a decrease in the induction of c-Fos and c-Myb [94]. Brown III et al. reported that homocysteine activates the mitogen-activated protein kinase (MAPK) signal transduction pathway, leading to an increase in cell proliferation and induction of the gene transcription factor c-Fos in VSMCs [95]. MicroRNA-217 alleviates the proliferation and migration of VSMCs induced by homocysteine through the targeted silencing of NMDARs and subsequent inhibition of the cAMP-response element binding protein/peroxisome proliferator-activated receptor γ coactivator 1α signaling pathway [96]. Beyond NMDARs activation, homocysteine also evokes oxidative and ER stress; the studies cited above provide varying levels of evidence and should be interpreted cautiously until endothelial-specific GRIN1 knock-out or peripherally restricted NMDARs blockers are used to conclusively separate receptor-mediated from non-receptor-mediated toxicity.

#### NMDARs and vasoconstriction or vasodilation

Vasoconstriction and vasodilation are two opposing physiological processes that regulate the diameter of blood vessels and affect blood flow and pressure. Vasoconstriction, the narrowing of blood vessels, occurs when the VSMCs of the vessel walls contract. Prolonged or inappropriate vasoconstriction can contribute to hypertension, Raynaud’s phenomenon, and other vascular diseases. In contrast, vasodilation, the widening of blood vessels, occurs when the VSMCs of the vessel walls relax, often in response to various stimuli, such as the release of NO. Excessive vasodilation can result in hypotension and shock [97, 98]. Endothelial dysfunction results in an altered balance between vasodilatory and vasoconstrictive mediators, along with an increased production of chemoattractant molecules and growth factors. In hypercholesterolemia, endothelial dysfunction accelerates the formation of fatty streaks, which are the earliest precursors of atherosclerotic plaques.

NMDARs activation in VSMCs regulates vascular function through several mechanisms. First, the activation of these receptors increases blood pressure and vascular resistance by promoting vasoconstriction. This effect may be relevant in conditions such as hypertension, in which excessive vasoconstriction contributes to elevated blood pressure [99]. However, NMDARs activation can also promote vasodilation, counteracting its vasoconstrictive effects. Overall, NMDARs activation alters the contractile function of VSMCs, leading to changes in blood flow and pressure, which may contribute to the development and progression of cardiovascular disease. For example, McGee et al. reported that the activation of peripheral vascular NMDARs leads to a dose-dependent increase in the mean arterial pressure in rats after the systemic infusion of NMDA. This effect was not affected by ganglion blockade (using hexamethonium) and was mitigated by NMDARs antagonists [14]. Second, nicotinamide adenine dinucleotide phosphate oxidase 2 (NOX2) is the major source of ROS during activation of NMDARs. nNOS-derived NO is the signaling molecule linking NMDARs to NOX2-dependent ROS production, which are important mediators of NMDARs -induced oxidative stress and cell damage, respectively [67]. The vasodilatory effect of NMDARs is mediated by the stimulation of NO production, which can relax VSMCs and decrease vascular resistance. This effect may be particularly important when vasodilation is beneficial, such as for the regulation of blood flow during exercise or in the treatment of vascular diseases. eNOS, expressed in VSMCs and endothelial cells, contributes to NO signal transduction. NO produced by vascular eNOS plays a crucial role in subsequent ROS generation and peripheral NMDAR-mediated pressor response [57, 100]. 

The mechanisms underlying the effects of NMDARs activation in VSMCs involve complex intracellular signaling cascades. NMDARs activation triggers a series of events that activate MAPKs and NF-κB. MAPKs are involved in cell proliferation, whereas NF-κB plays a crucial role in inflammation, which is a key component of atherosclerosis [101, 102]. NF-κB can increase vascular tension, promote vasoconstriction, and lead to elevated blood pressure by increasing the expression of endothelin-1, a potent vasoconstrictor [103]. However, NF-κB can also promote vasodilation by regulating the production of vasodilator factors, such as NO [104]. Pang et al. reported that homocysteine stimulates the NMDARs/ROS/MAPK/NF-κB signaling pathway, which leads to the upregulation of C-reactive protein expression in rat VSMCs. This initiates an inflammatory response in VSMCs and contributes to the development of atherosclerosis [105]. In rat VSMCs (A7r5), NMDARs can participate in homocysteine uptake, thereby stimulating this pathway and accelerating VSMCs dysfunction [106]. 

This paradoxical function is highly dependent on the cellular context and the specific signaling pathways activated upon receptor stimulation. The primary mechanism underlying both outcomes is the NMDARs-mediated influx of Ca²⁺, but the downstream consequences of this Ca²⁺ influx diverge significantly depending on the cell type. In VSMCs, an increase in intracellular Ca²⁺ concentration is a direct trigger for contraction. The binding of glutamate to NMDARs on VSMCs leads to Ca²⁺ influx, which activates calmodulin and subsequently myosin light chain kinase (MLCK). This enzyme phosphorylates myosin light chains, initiating the cross-bridge cycle between actin and myosin filaments and causing the muscle cell to contract, thereby narrowing the blood vessel lumen [7, 107]. This pathway is a key contributor to NMDARs-mediated vasoconstriction, particularly in peripheral vessels and under pathological conditions like pulmonary arterial hypertension [108]. Conversely, in endothelial cells, the same NMDARs-mediated Ca²⁺ influx can initiate a vasodilatory response. The increased Ca²⁺ in endothelial cells activates eNOS, the enzyme responsible for NO [109]. NO is a potent vasodilator that diffuses into the adjacent smooth muscle cells, where it activates soluble guanylyl cyclase (sGC) to produce cyclic guanosine monophosphate (cGMP). This second messenger activates protein kinase G (PKG), which promotes vasorelaxation through several mechanisms, including the stimulation of Ca²⁺ reuptake into the sarcoplasmic reticulum and the activation of large-conductance calcium-activated potassium (BK) channels, leading to membrane hyperpolarization and smooth muscle relaxation [110]. This dual role highlights NMDARs as versatile and critical regulators of vascular homeostasis, capable of fine-tuning blood flow in response to a myriad of physiological and pathological stimuli. Besides, the specific subunit composition of NMDARs can differ significantly between endothelial cells and VSMCs, contributing to their divergent roles in vascular tone regulation. In endothelial cells, the presence of GluN2C and/or GluN2D subunits may be more prominent. These subunits are less sensitive to Mg²⁺ blockade, which would allow the receptors to be more readily activated at the relatively hyperpolarized resting membrane potential of endothelial cells, facilitating a sustained Ca²⁺ influx and promoting eNOS-dependent vasodilation [111]. In contrast, NMDARs on VSMCs, particularly in the pulmonary circulation, appear to be enriched in GluN2A and GluN2B subunits. These subunits form receptors with higher Ca²⁺ permeability and stronger Mg²⁺ sensitivity, which could contribute to a more robust and transient Ca²⁺ influx upon activation, favoring a pro-constrictive response [7, 112]. The ultimate effect on vessel tone is therefore not a simple function of NMDARs activation but a complex integration of signals from different cell types within the vascular wall.

In summary, the relationship between NMDARs and VSMCs is complex and multifaceted, with significant implications for cardiovascular health and disease. A better understanding of this relationship may lead to the development of novel therapeutic strategies to prevent or treat cardiovascular diseases. (Fig. 3)

### NMDARs and endothelial cells

#### Overview

Endothelial cells line the interior surface of blood vessels and are responsible for the production of NO, which regulates blood pressure and flow. Endothelial dysfunction in predisposed regions of the arterial vascular system is an important factor in the pathophysiology of atherosclerotic cardiovascular diseases [113]. Characterized by impaired blood vessel dilation, increased oxidative stress, inflammation, leukocyte adhesion, and excessive vascular wall permeability, endothelial dysfunction allows LDL cholesterol and immune cells to easily infiltrate the vascular wall and promote atherosclerotic plaque formation [114, 115]. In peripheral tissues, NMDARs are expressed in endothelial cells and contribute to vascular function (Table 1). NMDARs activation in endothelial cells leads to the production of ROS and activation of signaling cascades that regulate vascular tone and blood pressure. These receptors participate in blood pressure regulation as well as regulate the function and morphology of endothelial cells and angiogenesis, thereby affecting blood vessel permeability and vasoconstriction. The functions of NMDARs in endothelial cells are listed in Table 1.

#### NMDARs and endothelial cell permeability

Endothelial cell permeability is a critical factor in the development of atherosclerosis. Under normal conditions, the endothelium acts as a selective barrier preventing the passage of large molecules and cells from the blood into the vessel wall. However, when endothelial cells become dysfunctional, their permeability increases, which leads to a series of events that contribute to atherosclerosis, including inflammation, smooth muscle cell proliferation, and plaque formation [116]. By modulating the activity of tight junction proteins and cytoskeletal elements, NMDARs activation can regulate endothelial cell permeability, thereby affecting vascular barrier function. This process is crucial for the maintenance of vascular homeostasis and prevention of vascular leakage. The activation of NMDARs by Glu, a major excitatory neurotransmitter in the CNS, leads to an influx of calcium into endothelial cells that line the blood–brain barrier (BBB), resulting in cytoskeletal rearrangements and increased paracellular permeability [117, 118]. These events ultimately increase BBB permeability, allowing larger molecules and circulating immune cells to enter the brain [119]. At high concentrations of Glu, NMDARs are overactivated in cerebral endothelial cells, leading to a significant increase in ROS formation. This increase is facilitated by the increased calcium ion influx through the receptor, resulting in endothelial dysfunction [120]. 

Unlike the CNS where the blood-brain barrier tightly regulates glutamate access, the peripheral vasculature lacks such strict compartmentalization. Consequently, extracellular glutamate in the vascular microenvironment can activate NMDARs on local cells through paracrine and autocrine mechanisms, making local synthesis and release particularly relevant to atherosclerosis. The development of atherosclerosis begins with the apoptosis and dysfunction of vascular endothelial cells. NMDARs overactivation in endothelial cells caused by homocysteine can lead to excessive ROS production and calcium influx. This results in the accumulation of asymmetric dimethylarginine (ADMA), a competitive inhibitor of the normal eNOS substrate, l-arginine. ADMA inhibits eNOS activity and reduces NO production [121], while decreasing the activity of the l-arginine transporter CAT-1 in aortic endothelial cell [122]. These changes ultimately contribute to endothelial cell apoptosis and vessel wall damage (Fig. 4).

#### NMDARs and angiogenesis

NMDARs activation in endothelial cells plays a role in angiogenesis and vascular repair by promoting endothelial cell migration, proliferation, and tube formation [108, 123]. Chen et al. reported that all subunits of NMDARs are expressed in rat aortic endothelial cells. They also found that homocysteine-induced NMDAR activation upregulates NMDAR1 expression and promotes aortic endothelial cell proliferation [124]. Flupirtine, an indirect NMDARs antagonist derived from triaminopyridine, aids in neuroprotection and neuro-angiogenesis following ischemic events by reducing the activation of calcium-induced JNK and NF-κB signaling pathways, which are linked to decreased proteasome activity, eventually helping reduce ischemic brain injury [125]. A recent study has indicated that the activation of NMDARs triggers ferroptosis, a type of regulated cell death involving iron-dependent lipid peroxidation, in endothelial cells through the PP2A-AMPK-HMGB1 signaling pathway [126]. These findings have potential implications for angiogenesis (Fig. 4).

Preserving the integrity of the endothelial barrier is crucial to prevent the onset and progression of atherosclerosis. Consequently, additional research is required to clarify the specific mechanisms through which NMDARs affect endothelial cell function, including their effects on endothelial permeability and angiogenesis, and its broader implications for vascular diseases. Targeting NMDARs in endothelial cells may provide innovative therapeutic avenues for the treatment of cardiovascular diseases.

## NMDARs and inflammation

Inflammation is a vital biological response that enables the body to defend itself against harmful stimuli such as pathogens, damaged cells, or irritants [127]. However, excessive or chronic inflammation can lead to tissue damage and contribute to the development of several diseases, such as cardiovascular diseases, cancer, diabetes, and neurological diseases. Inflammation plays a crucial role in the development and progression of atherosclerosis. When the vascular wall is stimulated by various factors, such as hypertension, hyperlipidemia, and smoking, the blood vessels are damaged, which triggers an inflammatory response [128, 129]. Recent research has highlighted the intricate relationship between NMDARs and inflammation, revealing their potential role in atherosclerosis. Specifically, homocysteine, monocyte chemoattractant protein-1 (MCP-1), and other inflammatory and chemotactic factors play crucial roles in the pathogenesis of atherosclerosis by promoting inflammatory responses, oxidative stress, and the recruitment and activation of immune cells. For instance, Zeng et al. have shown that homocysteine increases vascular inflammation by promoting the expression and secretion of MCP-1 and IL-8 in human monocytes in vitro [130]. In addition, Dai et al. demonstrated that homocysteine triggers the secretion of MCP-1 through oxidase-generated ROS in monocytes and macrophages [131]. Zanin et al. determined that homocysteine stimulates the synthesis and secretion of IL-1β in mouse macrophages through NF-ĸB, ERK, and P2 × 7 [132]. Moreover, Holven et al. reported that supplementation with folate (FA), a B vitamin necessary for basic biological functions, reduces the release of MCP-1 and IL-8 from peripheral blood mononuclear cells in patients with hyperhomocysteinemia [133]. Zhong et al. reported that aerobic exercise (treadmill training) and FA supplementation synergistically attenuate homocysteine levels and atherosclerotic plaques [134]. Overall, homocysteine appears to play a significant role in promoting vascular inflammation and atherosclerosis through various mechanisms, including the induction of chemotactic factors and inflammatory cytokines. It should be noted that homocysteine also evokes oxidative and ER stress independently of NMDARs, Future work should use endothelial-specific GRIN1-knock-out or peripherally restricted NMDARs blockers to provide definitive evidence of NMDARs involvement.

NMDARs activation in the microglia significantly contributes to cortical injury by regulating inflammation. This may accelerate the progression of neurological diseases by inducing microglial activation and the secretion of inflammatory factors that lead to cortical neuronal cell death and neurotoxicity [135]. Propofol, an intravenous hypnotic drug, can effectively mitigate LPS-induced microglial inflammation by inhibiting NMDARs-mediated Ca^2+^ influx, which subsequently leads to the decreased phosphorylation of calcium/calmodulin-dependent protein kinase II, ERK1/2, and NF-κB. This inhibition of phosphorylation effectively suppresses the activation of inflammatory signaling pathways [136]. Lebrun et al. reported that inhibiting endothelial NMDARs signaling effectively alleviates ischemic damage in a mouse model of thromboembolic stroke with chronic hyperglycemia and improves stroke outcomes in hyperglycemic mice by reducing inflammation and hemorrhage [137]. The NMDARs antagonist MN-08, a novel nitrate derivative of memantine, mitigates LPS-induced injury and inflammatory cell infiltration in mice through the inhibition of MAPK signaling pathway activation and STAT3/NF-κB signaling transduction, thereby improving systemic inflammatory response syndrome and immune dysfunction [138]. Considering the role of NMDARs in microglial activation and inflammatory responses in the context of cortical damage and neurodegenerative diseases, along with the potential therapeutic benefits of NMDARs inhibitors, the study of the relationship between NMDARs, inflammation, and atherosclerosis has entered a new swift developmental phase. Further research into the mechanisms underlying this interplay may provide insights into the development of inflammatory and cardiovascular diseases and potential treatment avenues.

## NMDARs and lipid metabolism

### Overview

Recent research has uncovered an unexpected link between NMDARs and lipid accumulation, a process that is closely associated with the development of metabolic diseases, such as obesity, diabetes, atherosclerosis, and cardiovascular diseases. NMDARs activation by Glu, the primary neurotransmitter that binds to NMDARs, stimulates lipid synthesis and accumulation in various cell types. NMDARs also appear to be involved in the regulation of lipid rafts.

### NMDARs and lipid synthesis and accumulation

Recent studies have shown that NMDARs may interact with signaling pathways that regulate lipid synthesis and metabolism. For example, Schumacher et al. reported that neurotransmitter receptors, particularly type A γ-aminobutyric acid receptors, NMDARs, and sigma 1 receptors, are involved in the biosynthesis of neurosteroids, which are synthesized de novo in the CNS from cholesterol and exert protective effects through their anti-convulsant, anti-anxiety, and anti-depressant activities [139]. In addition, NMDARs may affect the expression of genes that regulate lipid metabolism. For instance, Huang et al. reported that NMDARs activation induces lipid accumulation and hepatic steatosis through the ERK1/2/PPARα pathway in hepatocytes [140]. NMDARs activation enhances the degradation of ABCA1 protein through a calcium-activated protease, resulting in a decrease in ABCA1 protein on the cell membrane surface. This process promotes lipid accumulation in macrophages and stimulates the production and secretion of inflammatory mediators [70]. NMDARs are key players in neuronal excitability and synaptic plasticity and seemingly play significant roles in lipid metabolism and neurosteroid synthesis. The modulation of these pathways by NMDARs may inspire novel treatments for conditions related to the dysregulation of lipid metabolism and atherosclerosis.

Lipid rafts, specialized membrane microdomains enriched in cholesterol, sphingolipids, and various proteins, play crucial roles in cellular processes, such as signal transduction, membrane trafficking, and cell adhesion [141]. NMDARs are localized in lipid rafts within the cell membrane, where they interact with other proteins and signaling molecules to form signaling complexes [142]. This localization is essential for NMDARs function because lipid rafts provide a unique environment conducive to receptor clustering, signal transduction, and interactions with downstream effectors [143]. Although previous research has demonstrated the existence of NMDARs subclasses within lipid rafts, the molecular link between NMDARs and lipid raft proteins remains unclear. Lipid rafts also play a regulatory role in NMDARs function. Previous research has shown that NMDARs interact with lipid raft-affiliated proteins, with different GluN2 subunits exhibiting distinct binding patterns. Specifically, GluN2B subunits directly bind to both flotillin 1 and 2, whereas GluN2A subunits directly bind to only flotillin 1 [144]. Flotillins recruit NMDARs into lipid rafts, where they initiate secondary messenger signaling cascades to activate the intracellular calcium signaling pathway. However, whether lipid rafts can regulate the NMDARs-mediated calcium pathway in atherosclerosis-related cells or even cause atherosclerosis is not clear and further studies are needed to confirm this.

## Compounds affecting NMDARs: Agonists vs. Antagonists

The structural features of agonist binding are conserved across all GluN subunits, with only a few agonists exhibiting subunit selectivity. However, some key differences do exist, which enable the design of subunit-selective ligands. Recently, the review by Hansen et al. (2021) provided a more detailed understanding of over three decades of research on ionotropic glutamate receptors (iGluRs), with a focus on the progress made in the past decade [44]. Building on this foundation, we will briefly introduce some pharmacological tools that can be used for studying NMDARs outside the CNS.

### Agonists

The GluN1/GluN2 NMDA receptors are distinguished from other iGluRs by their unique requirement for the simultaneous binding of two different agonists to achieve activation: glycine or D-serine to the GluN1 subunit, and glutamate to the GluN2 subunit [145]. To date, several other GluN2 subunit agonists have been identified, including: (a) endogenous agonists, such as D- and L-aspartate, homocysteic acid, and cysteic acid; (b) natural products, such as ibotenic acid (or ibotenate) isolated from the poisonous mushroom Amanita ibotengutus; and (c) glutamate analogs, such as L-CCG-IV, trans-ACBD, cis-ACPD, and quinolinic acid [146–149]. Most partial agonists also exhibit differences in agonist efficacy among the GluN2 subunits, which is attributed to interdomain interactions between the ABD and NTD, a finding that has been confirmed by studies of crystal and cryo-EM structures [150–152]. Agonists with some degree of GluN2 subunit selectivity in terms of agonist efficacy have been identified, including: (a) High efficacy at GluN1/2A; (b) High efficacy at GluN1/2B; (c) High efficacy at GluN1/2 C; (d) High efficacy at GluN1/2D [153, 154]. For example, ethyl-NHP5G and propyl-NHP5G exhibit very low efficacy at GluN1/2A, but high efficacy at GluN1/2D [155, 156]. Notably, (RS)-AMAA shows moderate efficacy at GluN1/2B and GluN1/2 C, but low efficacy at GluN1/2A [18]. N-methyl-L-aspartate demonstrates moderate efficacy at GluN1/2B to GluN1/2Dm but very high efficacy at GluN1/2A. In contrast, N-methyl-D-aspartate (NMDA) shows high efficacy at all GluN1/2 subunits [18]. Interestingly, D-serine and glycine are endogenous co-agonists for the GluN1/2 NMDA receptors. However, D-cycloserine acts as a partial agonist at NMDA receptors containing GluN2A, GluN2B, and GluN2D; however, it serves as a superagonist at receptors containing GluN2C, with higher agonist efficacy than the endogenous agonist glycine. D-serine is also a superagonist at receptors containing GluN2C, although its potency is lower than that of D-cycloserine [157, 158]. The D- and L-isomers of glycine, alanine, and serine all act as GluN1 agonists. GluN3A and GluN3B also bind glycine and D-serine; however, certain differences exist between the agonist-binding regions of GluN3 and GluN1 subunits. Existing studies have confirmed that glycine binds to GluN1 in GluN1/2 receptors with high potency (0.1-1 µM), whereas it binds to GluN3A in GluN3A receptors with lower potency (10–60 µM) [159]. In addition, in GluN1/3 receptors, D-serine acts as a partial agonist [160] (Table 2).

**Table 2:** Compounds affecting NMDARs: Agonists vs. Antagonists

Type	Appellation	GluN1/2A	GluN1/2B	GluN1/2 C	GluN1/2D	GluN1/3A	GluN1/3B
agonist	*L*-glutamate	++++	++++	++++	++++	++++	++++
	*D*-glutamate	++++	++++	++++	++++	++++	++++
	Glycine	++++	++++	++++	++++	++++	++++
	*D-*aspartate	++++	++++	++++	++++	++++	++++
	*L*-aspartate	++++	++++	++++	++++	++++	++++
	*D*-serine	++++	++++	++++	++++	++++	++++
	*D*-glycine	++++	++++	++++	++++	++++	++++
	N-methyl-L-aspartate	++++	+++	+++	+++	+++	+++
	N-methyl-D-aspartate	++++	++++	++++	++++	++++	++++
	*D*-cycloserine	++++	+++	++++	++++	NA	NA
	*L*-homocysteic	++++	++++	+++	+++	NA	NA
	*D*-homocysteic	++++	++++	+++	++++	NA	NA
	cysteic	++++	++++	++++	++++	NA	NA
	ibotenic	++++	++++	+++	++++	NA	NA
	*L*-CCG-IV	++++	++++	++++	++++	NA	NA
	trans-ACBD	++++	+++	++++	++++	NA	NA
	cis-ACPD	+++	++	++	+++	NA	NA
	ethyl-NHP5G	+	++	++	+++	NA	NA
	propyl-NHP5G	-	+	+	++	NA	NA
	(RS)-AMAA	+	+++	++	+++	NA	NA
Competitive Antagonists	7-CKA	++++	++++	NA	NA	NA	NA
	DCKA	++++	++++	++++	++++	NA	NA
	CGP 58,411	++++	++++	NA	NA	NA	NA
	ACEA-1-21 (licostinel)	++++	++++	++++	++++	NA	NA
	TK13	NA	NA	NA	NA	++	++
	TK30	NA	NA	NA	NA	++	++
	TK40	NA	NA	NA	NA	+++	+++
	TK80	NA	NA	NA	NA	++	++
	CGS 19,755 (selfotel)	++++	++++	++++	++++	NA	NA
	PMPA	++++	+++	+++	+++	NA	NA
	D-CPP	++++	++++	++++	+++	NA	NA
	NVP-AAM007	++++	++++	++++	++++	NA	NA
	ST3	++++	++++	++++	++++	NA	NA
	UBP791	+++	++++	++++	++++	NA	NA
	UBP170	++++	++++	++++	++++	NA	NA
	Con-G	++	++++	++	++	NA	NA
	Con-Pr3	++	++++	++	++	NA	NA
	Con-RIB	++	++++	++	++	NA	NA
	Con-Br	++	++	++	++++	NA	NA
	Con-Bk-C	++	++	++	++++	NA	NA
Voltage-Dependent Channel Blockers	ketamine	+++	+++	++++	++++	+++	+++
	phencyclidine (PCP)	++++	++++	++++	++++	++++	++++
	MK-801	++++	++++	+++	+++	+++	+++
	amantadine	++	++	++	++	++	++
	memantine	+++	+++	++++	++++	+++	+++
	dextromethorphan	+++	+++	+++	+++	+++	+++
	dextrorphan	++++	++++	++++	++++	++++	++++
	lanicemine (AZD6765)	+++	+++	++	++	++	++
	N1 dansyl spermine	++	++	++	+++	++	++
	tribenzylamine TB-3-4	++	++	++	+++	++	++

L-CCG-IV, Cyclopropaneaceticacid, a-amino-2-carboxy-,(aS,1R,2 S)-; trans-ACBD, 1-aminocyclobutane-r-1,cis-3-dicarboxylic acid; cis-ACPD, cis-(+/-)-1-aminocyclopentane-1, 3-dicarboxylic acid; NHP5G, N-hydroxypyrazole-5-glycine; (RS)-AMAA, (RS)-2-amino-2-(3-hydroxy-5-methyl-4-isoxazolyl)acetic acid; 7-CKA, 7-chlorokynurenic acid; DCKA, 5,7-dichlorokynurenic acid; ACEA 1021, 6,7-dichloro-5-nitro-1,4-dihydro-2,3-quinoxalinedione; TK13, [2-hydroxy-5-((4-(pyridin-3-yl)thiazol-2-yl)amino]benzoic acid; TK30, 4-(2,4-dichlorobenzoyl)-1 H-pyrrole-2-carboxylic acid; TK40, 1-thioxo-1,2-dihydro- [1, 2, 4]triazolo[4,3-a]quinoxalin-4(5 H)-one; TK80, 6-hydroxy- [1, 2, 5]oxadiazolo[3,4-b]pyrazin-5(4 H)-one; PMPA, hosphonomethylpentanedioic acid; CGS-19755, cis-4-(phosphonomethyl)-2-piperidine-carboxylic acid; D-CPP, D(-)3-(2-carboxypiperazin-4-yl)-propenyl-l-phosphonic acid; NVP-AAM007, (S)-5-[(R)-2-amino-2-carboxyethyl]-1-[4-(3-fluoropropyl)phenyl]-4,5-dihydro-1 H-pyrazole-3-carboxylic acid; Con-G, Conantokin-G; MK-801, dizocilpine maleate; UBP791, (2 S*,3R*)-1-(7-(2-carboxyethyl)phenanthrene-2-carbonyl)piperazine-2,3-dicarboxylic acid; UBP1700, 2 S*,3R*)-1-(7-(2-carboxyvinyl)phenanthrene-2-carbonyl)piperazine-2,3-dicarboxylic acid; The more “+” signs, the stronger the effect; “-“, no effect; NA, no data are available. (For more details, refer to the recent review by Hansen et al. 2021) [44]

### Competitive antagonists

Most competitive GluN1 antagonists can be primarily divided into two major categories. The first category includes high-affinity antagonists such as 7-chlorokynurenic acid (7-CKA), 5,7-dichlorokynurenic acid (DCKA), L-683,344, L-689,560, L-701,324, and GV150526A (gavestinel). The second category includes CGP 78,608 and ACEA-1021. Among these, 7-CKA, L-689,560, L-701,324, and CGP 78,608 exhibit higher selectivity for the GluN1/2A and GluN1/2B subunits [44]. Due to the complex activation characteristics of GluN1/3 receptors, only a few studies have described competitive GluN3 antagonists to date [159, 161, 162]. The competitive antagonists TK40 and DCKA exhibit approximately 80-fold and 500-fold higher affinity for the GluN1 subunit compared to the GluN3 subunit, respectively [161]. In addition, TK13, TK30, and TK80 are competitive antagonists for GluN1/3 receptors, although they have relatively lower potency and moderate selectivity [162]. Because the agonist-binding regions of GluN2 NMDA receptor subunits are highly conserved, no competitive antagonists with high selectivity for a single GluN2 subunit have been developed to date. The earliest developed GluN2 competitive antagonists are (R)-AP5 (or D-APV) and (R)-AP7 [163]. From these, a series of AP5 and AP7 analogs have been developed, including CGS 19,755 (selfotel), PMPA, D-CPP, D-CPPene, LY235959, NPC 17,742, and SDZ 220 − 040 [163]. In addition, NVP-AAM007 (or PEAQX), which has high affinity, still lacks sufficient GluN2 subunit selectivity to block only GluN2A receptors without blocking GluN2B receptors [164, 165]. Presently, there are better selective pharmacological tools to dissect the physiological roles of GluN2 subunits. A series of 3-carboxy-pyrazolino amino acids serve as competitive antagonists with binding preferences among GluN2 subunits. For example, ST3 has a 15-fold preference for GluN2A over GluN2B, but has moderate affinity for GluN2C and GluN2D subunits [44]. Moreover, high-affinity antagonists such as UBP791 and UBP1700 show a 15- to 40-fold preference for GluN2C/D over GluN2A/B subunits [166]. Peptides derived from the venom of predatory marine snails (Conus snails), known as conantokins and their derivatives, also exhibit better subunit selectivity. For instance, Con-G, Con-Pr3, and Con-RIB preferentially inhibit NMDA receptors containing GluN2B alone, whereas Con-Br and Con-Bk-C are more effective at inhibiting NMDA receptors containing GluN2D [167, 168]. These competitive inhibitors offer a better pharmacological toolkit for NMDARs researchers. (Table 2)

### Voltage-dependent channel blockers

Channel blockers are a class of compounds that inhibit the function of ion channels by physically obstructing the channel pore, preventing ion flow. These blockers can be highly specific, targeting particular ion channels such as NMDARs [169]. NMDA receptor channel blockers are primarily divided into two major categories: (a) high-affinity NMDARs channel blockers, such as ketamine, phencyclidine (PCP), and MK-801; and (b) low-affinity NMDARs channel blockers, such as amantadine, memantine, dextromethorphan, and its metabolite dextrorphan, as well as lanicemine (AZD6765) [44]. Due to the relative conservation of the channel pore within NMDA receptors, channel blockers exhibit only moderate subunit selectivity for the GluN2 subtypes. For example, MK-801 has 10-fold higher efficacy at receptors containing GluN2A and GluN2B compared to those containing GluN2C and GluN2D [170]. In the presence of physiological Mg²⁺, memantine and ketamine show 10-fold selectivity for receptors containing GluN1/2 C and GluN1/2D over those containing GluN1/2A and GluN1/2B [171, 172]. In addition, aryl polyamine derivatives such as N1 dansyl spermine and tribenzylamine TB-3-4 have 40-fold higher efficacy at GluN2D-containing NMDA receptors compared to those containing GluN2A [173]. It is important to emphasize that channel blockers remain valuable tools in both preclinical and clinical research. The appropriate selection of channel blockers is crucial, especially to avoid using them at high concentrations, which can significantly undermine confidence in the observed biological effects being specifically related to NMDARs (Table 2).

## NMDARs antagonists in atherosclerosis

NMDARs exert a wide range of effects on abnormal lipid metabolism in macrophages, inflammatory responses, vascular function, endothelial dysfunction, and smooth muscle cell proliferation. This may explain the mechanisms through which these receptors affect the development of atherosclerosis. NMDARs antagonists have shown promising potential for the treatment of neurodegenerative diseases and been effectively utilized in therapeutic investigations across various disease models [174, 175]. The previous section provides a detailed introduction to the classification and selection of NMDA antagonists. Building on this foundation, subsequent research should further explore the clinical applications and potential therapeutic significance of these agents, particularly in the context of cardiovascular diseases. This would involve investigating their efficacy in treating conditions such as atherosclerosis, hypertension, and other cardiovascular disorders, as well as evaluating their safety and optimal dosing regimens through well-designed clinical trials.

The direct therapeutic link between NMDARs antagonists and atherosclerosis remains unclear. However, recent studies have illuminated a profound connection between NMDARs antagonists and several pivotal processes that contribute to the progression of atherosclerosis, such as the inflammatory response, oxidative stress, and apoptosis. These findings provide novel perspectives on the pathological mechanisms underlying atherosclerosis and may facilitate the discovery of innovative therapeutic approaches. For example, Wu et al. reported that a novel memantine nitrate derivative, MN-08, exhibits a distinctive dual-action mechanism. This compound prevents calcium ion overload in neural cells by blocking NMDARs and stimulates the release of NO to dilate blood vessels, thereby enhancing cerebral blood flow [176]. In addition, Hao et al. demonstrated that the NMDARs blocker memantine effectively mitigates the inflammatory response, oxidative stress, and apoptosis induced by oxLDLs in human umbilical vein endothelial cells through the activation of the BDNF/TrkB signaling pathway [177]. In a recent study, Clemmensen et al. developed a novel therapeutic agent that integrates GLP-1 analogs with the NMDARs antagonist MK-801. This innovative compound has demonstrated significant efficacy against obesity, hyperglycemia, and dyslipidemia in rodent models of metabolic disorders. The mechanism underlying its therapeutic effects involves the modulation of neuroplasticity within the hypothalamus and brainstem [178]. During ischemic diseases and reperfusion, intracoronary administration of NMDARs antagonists MK-801, memantine, and ifenprodil reduces the production of ROS and alters cardiac function, thereby alleviating myocardial cell apoptosis and mitigating damage to the myocardium [179, 180]. A prior retrospective cohort analysis demonstrated that the NMDARs antagonist amantadine can reduce adverse cardiovascular and cerebrovascular events and lower the risk of heart failure and arrhythmias [17]. Currently, there is no conclusive evidence that NMDARs antagonists exhibit therapeutic benefits in atherosclerosis, necessitating future research. These preliminary studies present a novel viewpoint on the interplay between NMDARs antagonists and atherosclerosis, indicating promising novel avenues for future exploration. As research progresses, we anticipate deciphering the precise mechanisms underlying these interactions and developing innovative therapeutic approaches for the management of atherosclerosis.

## Current challenges

A major issue is the poor selection of pharmacological reagents used by labs studying peripheral receptors. Typically, non-selective and low-potency agents, several dating back 20–30 years, are used. This raises concerns regarding whether the observed biological effects are truly related to NMDARs. Examples include early low-affinity agonists, channel blockers such as memantine, and even MK801. In addition, these agents are often used at high concentrations, which further undermines the reliability of some findings. Following the cloning of various GluN subunits, it became feasible to quantitatively assess the activity of these ligands at recombinant GluN subtypes expressed in heterologous systems. This method enabled the discovery of new classes of orthosteric ligands and channel blockers with diverse chemical structures, some exhibiting subunit selectivity (reviewed by Hansen 2021) [44]. More recently, advancements in X-ray crystallography and molecular dynamics have offered deeper insights into binding mechanisms and the structural basis for subunit selectivity [181, 182]. This will help provide guidance in the NMDARs field with regard to selecting newly, highly efficient, and highly subunit-selective compounds for both clinical and basic research. Moreover, considering the critical role of NMDARs in the CNS and their well-established connections to neurodegenerative diseases, researchers focusing on peripheral glutamate receptors urgently need to develop tools that are restricted to peripheral action and do not affect the brain. Achieving this will require significant efforts to optimize these compounds so that they do not cross the blood–brain barrier. It is important to emphasize that several NMDARs agonists, such as homocysteine, have low efficacy and often exhibit actions unrelated to NMDARs. Therefore, researchers need to scrutinize the observed biological effects more carefully and validate them using more potent agonists.

## Conclusions and future perspectives

The role of NMDARs in atherosclerosis has recently gained significant research interest. Although NMDARs are primarily known for their function in the CNS, their presence and activity in non-neuronal tissues, including the heart, vasculature, macrophages, VSMCs, and endothelial cells have been increasingly recognized. As described in this review, several studies have now explored the effects of NMDARs on abnormal lipid metabolism in macrophages, inflammatory responses, vascular tone, endothelial dysfunction, and smooth muscle cell proliferation, all of which are implicated in the occurrence and progression of atherosclerosis. Understanding the molecular mechanisms underlying the NMDARs-mediated regulation of atherosclerosis-related cells is still evolving, particularly in relation to its interaction with related signaling pathways. This could help identify specific therapeutic targets against atherosclerosis within the NMDARs signaling pathway. For example, current evidence indicates that targeting NMDARs will provide novel therapeutic strategies for the prevention and treatment of this complex disease. Therefore, the development of more effective and selective NMDARs inhibitors is crucial for translating these findings into clinical applications. Current NMDARs inhibitors, although promising, have limitations in terms of specificity and potential side effects. Systemic blockade of NMDARs with first-generation antagonists such as MK-801 or ketamine is limited by psychotomimetic and neurotoxic effects. Therefore, future therapeutic strategies should focus on peripherally restricted antagonists (e.g., charged derivatives or nanoparticle-encapsulated formulations) that spare central NMDARs function while suppressing peripheral plaque inflammation. Further, clinical trials to evaluate the efficacy and safety of NMDAR targeting in patients with atherosclerosis are warranted. In conclusion, NMDARs represent an exciting novel frontier in atherosclerosis research, with the potential to significantly advance our understanding of the disease and inspire the development of more effective therapeutic interventions. With further research and clinical validation, targeting NMDARs could emerge as a powerful tool against atherosclerosis and its devastating cardiovascular consequences.

## Methodology

During the early preparation phase of this project, we conducted a structured literature search with the following steps:

(1) Databases queried: PubMed, Embase, and Web of Science;

(2) Core keywords and Boolean strings: “N-Methyl-d-aspartate receptors AND atherosclerosis,” “NMDAR AND atherosclerosis,” “NMDAR AND macrophage,” “NMDAR AND vascular smooth-muscle cell,” “NMDAR AND endothelial cells,” “NMDAR AND inflammation,” etc.;

(3) Date range: January 1990 – September 2025;

(4) Inclusion/exclusion criteria: English-language, peer-reviewed articles reporting human or mammalian in-vivo or primary-cell data; conference abstracts, non-mammalian models, and purely structural studies were excluded.

## Data Availability

Not applicable.
